# Exploring the Role of Hemogram-Derived Ratios and Liver Fibrosis Scores in Pulmonary Fibrosis

**DOI:** 10.3390/medicina60101702

**Published:** 2024-10-16

**Authors:** Vera Ciornolutchii, Victoria Maria Ruta, Adina Milena Man, Nicoleta Stefania Motoc, Stefan-Lucian Popa, Dan L. Dumitrascu, Abdulrahman Ismaiel, Daniel-Corneliu Leucuta

**Affiliations:** 1 2nd Department of Internal Medicine, “Iuliu Hatieganu” University of Medicine and Pharmacy, 400006 Cluj-Napoca, Romania; veraciornolutchii11@gmail.com (V.C.); popa.stefan@umfcluj.ro (S.-L.P.); ddumitrascu@umfcluj.ro (D.L.D.); 2 Department of Pulmonology, “Leon Daniello” Clinical Hospital of Pulmonology, 400371 Cluj-Napoca, Romania; victoria.suteu@yahoo.com; 3 Department of Pneumology, “Iuliu Hatieganu” University of Medicine and Pharmacy, 400000 Cluj-Napoca, Romania; manmilena50@yahoo.com (A.M.M.); motoc_nicoleta@yahoo.com (N.S.M.); 4 Department of Medical Informatics and Biostatistics, “Iuliu Hatieganu” University of Medicine and Pharmacy, 400349 Cluj-Napoca, Romania; dleucuta@umfcluj.ro

**Keywords:** pulmonary fibrosis, idiopathic pulmonary fibrosis (IPF), secondary pulmonary fibrosis (SPF), hepatic steatosis, biomarkers, hemogram-derived ratios, liver fibrosis

## Abstract

*Background and Objectives:* Pulmonary fibrosis, including idiopathic pulmonary fibrosis (IPF) and secondary pulmonary fibrosis (SPF), is a progressive lung disease that significantly impairs respiratory function. Accurate differentiation between IPF and SPF is crucial for effective management. This study explores the association between pulmonary fibrosis and hepatic conditions, evaluating the utility of various hemogram-derived ratios and hepatic fibrosis scores in distinguishing between IPF and SPF. *Materials and Methods:* We conducted a retrospective study involving patients diagnosed with IPF or SPF at the “Leon Daniello” Clinical Hospital of Pneumology in Cluj-Napoca, Romania. Pulmonary fibrosis was confirmed via imaging techniques, and hepatic steatosis and fibrosis were assessed using non-invasive scores. We analyzed clinical, laboratory, and pulmonary function data, focusing on hemogram-derived ratios and hepatic scores. Statistical analyses, including ROC curves, were used to evaluate the effectiveness of these biomarkers in differentiating IPF from SPF. *Results:* We included a total of 38 patients with IPF and 28 patients with SPF. Our findings revealed that IPF patients had a significantly higher FIB-4 score compared to SPF patients, suggesting increased hepatic fibrosis risk in IPF, as well as an increased RDW/PLT ratio. Conversely, SPF patients exhibited elevated PLR, PNR, and SII, reflecting a more pronounced inflammatory profile. PLR and PNR demonstrated the highest discriminatory ability between IPF and SPF, while traditional hepatic fibrosis scores showed limited differentiation capabilities. No significant differences in pulmonary function tests were observed across hepatic fibrosis risk categories. *Conclusions:* The study highlights the value of biomarkers like PLR and PNR in differentiating between IPF and SPF, offering additional diagnostic insights beyond traditional imaging. Integrating hepatic assessments into the management of pulmonary fibrosis could improve diagnostic accuracy and patient care.

## 1. Introduction

Pulmonary fibrosis is a chronic and progressive interstitial lung disease characterized by the thickening and scarring of lung tissue, which leads to a decline in respiratory function [1]. Idiopathic Pulmonary Fibrosis (IPF) represents the most severe form of this condition, with a median survival rate of only 3 to 5 years following diagnosis [2]. It predominantly affects older adults and is slightly more common in men than women [3]. While IPF remains a disease of unknown etiology, Secondary Pulmonary Fibrosis (SPF) arises from known causes, including environmental exposures, autoimmune diseases, and certain medications [4]. Both IPF and SPF present significant clinical challenges, due to their progressive nature and the limited efficacy of current treatment options [5].

Differentiating between IPF and SPF is critical for patient management and prognosis. Current assessment methods primarily rely on high-resolution computed tomography (HRCT) to identify characteristic patterns of lung damage, such as the usual interstitial pneumonia (UIP) pattern, which is highly suggestive of IPF [6]. However, in cases where the HRCT findings are inconclusive or where the lung biopsy is not feasible, distinguishing between IPF and SPF can be challenging [7]. In these instances, additional diagnostic tools and biomarkers are often necessary to accurately categorize the type of pulmonary fibrosis and guide appropriate treatment strategies [8,9].

Patients with pulmonary fibrosis frequently present with multiple comorbidities that complicate their clinical management [10]. Cardiovascular diseases, including pulmonary hypertension, are common, as are conditions like gastroesophageal reflux disease (GERD) and sleep apnea [11,12,13]. The presence of these comorbidities often worsens the patient’s overall prognosis and contributes to the complexity of their care [14]. Understanding the full spectrum of associated conditions is essential for the holistic management of patients with pulmonary fibrosis.

Emerging evidence suggests a potential link between pulmonary fibrosis and hepatic disorders, particularly hepatic steatosis and liver fibrosis [15]. These conditions may share common pathophysiological mechanisms, including chronic inflammation and fibrogenesis, which could contribute to their co-occurrence [16,17]. Non-invasive scores and biomarkers have been developed to assess hepatic steatosis and fibrosis, offering a valuable tool for investigating their prevalence and impact in patients with pulmonary fibrosis [18]. This association may have implications for the severity of pulmonary involvement and overall disease progression [19].

The current study aims to evaluate the association between pulmonary fibrosis, both idiopathic and secondary, diagnosed using pulmonary CT, and hepatic steatosis and fibrosis assessed through non-invasive scores and biomarkers. We also seek to explore the utility of hemogram-derived ratios and other biomarkers in distinguishing between IPF and SPF. Furthermore, we examined the relationship between hepatic conditions and pulmonary function tests to assess whether hepatic involvement predicts more severe pulmonary disease.

## 2. Methods

### 2.1. Study Participants

This retrospective study was conducted at the “Leon Daniello” Clinical Hospital of Pneumology in Cluj-Napoca, Romania. The study included patients diagnosed with either IPF or SPF during their hospitalization. IPF was confirmed based on a typical pattern of UIP identified via native CT or HRCT, either with or without lung biopsy, provided there were no other identifiable causes of pulmonary fibrosis. SPF was confirmed by native CT or HRCT in patients who had identifiable underlying causes, including sarcoidosis or collagen vascular diseases. Patients with conditions such as tumors, hepatitis B or C, alcoholic liver disease, autoimmune hepatitis, drug-induced liver injury (DILI)/herb-induced liver injury (HILI), acute hemolytic diseases, acute inflammatory pathologies, and any liver disease were excluded. Additionally, patients with unclear pulmonary fibrosis diagnoses or missing data were not included in the study.

### 2.2. Pulmonary Fibrosis Assessment

IPF was defined as a specific form of chronic, progressive fibrosing interstitial pneumonia of unknown cause, occurring primarily in older adults. It was characterized by the histopathologic and/or radiologic pattern of UIP. Diagnosis was confirmed either by native CT or HRCT showing a typical UIP pattern, or through lung biopsy when imaging was inconclusive [20,21]. The UIP pattern included reticular opacities, often associated with honeycombing and traction bronchiectasis, typically with a subpleural and basal predominance. Exclusion criteria for IPF included any other identifiable cause of pulmonary fibrosis, such as heavy-metal exposure, drug-induced reactions, pulmonary irradiation, hypersensitivity pneumonitis, sarcoidosis, bronchiolitis obliterans, HIV infection, viral hepatitis, cancer, or collagen vascular diseases (e.g., scleroderma, polymyositis/dermatomyositis, systemic lupus erythematosus, rheumatoid arthritis).

SPF was defined as pulmonary fibrosis that occurs secondary to an identifiable cause, such as autoimmune diseases, chronic hypersensitivity pneumonitis, or exposure to environmental or occupational agents [22]. Diagnosis was confirmed by native CT or HRCT, revealing fibrotic changes consistent with known etiologies of pulmonary fibrosis. Underlying conditions that could lead to SPF, such as sarcoidosis or collagen vascular diseases (e.g., scleroderma, polymyositis/dermatomyositis, systemic lupus erythematosus, rheumatoid arthritis), were thoroughly investigated to establish the diagnosis.

### 2.3. Data Collection

Data were retrospectively collected from hospital records, covering several clinical and laboratory parameters.

#### 2.3.1. Laboratory Analysis

All laboratory analyses were performed using standardized methods in the hospital’s central laboratory. Blood samples were collected during routine clinical assessments, and the following tests were performed: Complete Blood Count (CBC), including measurements of white blood cell count, red blood cell count, hemoglobin levels, hematocrit, platelet count, and differential leukocyte count. These values were used to calculate hemogram-derived ratios. Liver Function Tests (LFTs) including aspartate aminotransferase (AST), alanine aminotransferase (ALT), alkaline phosphatase (ALP), and gamma-glutamyl transferase (GGT), were measured using automated biochemical analyzers. These enzymes were critical in calculating hepatic steatosis and fibrosis scores. Lipid-profile levels of total cholesterol and triglycerides were measured to assess metabolic status and to contribute to the calculation of certain hepatic indices. Renal function tests including creatinine and urea were assessed, and estimated Glomerular Filtration Rate (eGFR) was calculated. Fasting plasma sugar levels were measured to evaluate metabolic conditions which may be associated with hepatic steatosis. These laboratory data were essential for calculating non-invasive hepatic scores and assessing systemic inflammation, providing a basis for investigating their potential association with pulmonary fibrosis severity.

#### 2.3.2. Pulmonary Function Tests (PFTs)

Pulmonary function was assessed using standard spirometry, which measured several parameters including Forced Vital Capacity (FVC), Forced Expiratory Volume in one second (FEV1), FEV1/FVC, Maximal Expiratory Flow (MEF) 75%, MEF 50%, and MEF 25%, as well as Diffusing Capacity of the Lung for Carbon Monoxide (DLCO) [23]. These tests provided insight into the severity of lung impairment in patients with pulmonary fibrosis. The results were expressed according to the predicted normal values, adjusted for age, gender, height, and ethnicity.

#### 2.3.3. Hepatic Steatosis and Liver Fibrosis Scores

Hepatic steatosis and liver fibrosis were evaluated using non-invasive scores including the Fibrosis-4 Index (FIB-4), AST to Platelet Ratio Index (APRI), aspartate aminotransferase to alanine aminotransferase (AST/ALT) ratio, body mass index (BMI), AST/ALT ratio, and diabetes (BARD), age, bilirubin, INR and serum creatinine level (ABIC) score, King’s score for liver fibrosis, Logarithm of Odds for Lok Index (LogOddsLok), Lok Index for Liver Fibrosis (Lok index), and triglyceride to glucose (TyG) ratio [23,24]. These scores were calculated based on routinely available laboratory data and were used to assess the degree of hepatic involvement in the study population. Advanced fibrosis probability was calculated according to the current recommendations for each score, individually.

#### 2.3.4. Hemogram-Derived Ratios and Biomarkers

Several hemogram-derived ratios and systemic inflammation markers were assessed including Neutrophil-to-Lymphocyte Ratio (NLR), Derived Neutrophil-to-Lymphocyte Ratio (dNLR), Platelet-to-Lymphocyte Ratio (PLR), Lymphocyte-to-Monocyte Ratio (LMR), Eosinophil-to-Lymphocyte Ratio (ELR), Basophil-to-Lymphocyte Ratio (BLR), red cell distribution width (RDW-CV)-to Platelet ratio, and Systemic Immune–Inflammation Index (SII), a composite index calculated as (Platelet count × Neutrophil count)/Lymphocyte count, reflecting the balance between inflammation and immune response.

### 2.4. Statistical Analysis

Data were presented as means with standard deviations (SDs) for normally distributed quantitative variables, medians with interquartile ranges (IQRs) for non-normally distributed quantitative data, and as numbers with percentages for categorical variables. Clinical characteristics were compared between groups using appropriate statistical tests: a *t*-test for independent samples for normally distributed quantitative variables, the Wilcoxon rank-sum test for non-normally distributed quantitative variables, and χ2 tests or Fisher’s exact tests for categorical data. ROC analysis was employed to assess the accuracy of hepatic steatosis and liver fibrosis scores, as well as hemogram-derived ratios, in differentiating between IPF and SPF. A *p*-value of <0.05 was considered statistically significant. All analyses were performed using R software version 4.1.2 (R Foundation for Statistical Computing).

## 3. Results

### 3.1. General Characteristics

In this study, a comparative analysis between patients with IPF and SPF revealed several significant findings, as outlined in Table 1. The median age of patients with IPF was significantly higher, at 73 years, compared to 68 years in the SPF group (*p*-value = 0.021). A significantly higher proportion of males were present in the IPF group (60.53%) compared to the SPF group (32.14%) (*p*-value = 0.023). However, other variables, such as BMI and smoking history, did not show significant differences between the two groups. The inflammatory marker C-reactive protein (CRP) and liver function tests, including AST and ALT, also showed no significant differences. Notably, total bilirubin levels were higher in the IPF group compared to the SPF group, with a median of 0.74 mg/dL versus 0.52 mg/dL, respectively (*p*-value = 0.011). Other biochemical markers, such as ALP, GGT, and renal function tests, including urea, creatinine, and eGFR, showed no significant differences between the two groups. Additionally, lipid profiles and RDW-CV were comparable, indicating similar metabolic and hematological profiles between the IPF and SPF patients.

Most etiologies of SPF included rheumatoid arthritis (*n* = 9), as well as rheumatoid arthritis associated with antisynthetase syndrome (*n* = 1), COVID-19 infection (*n* = 1), RS3PE syndrome (*n* = 1), Sjogren syndrome and antisynthetase syndrome (*n* = 1), Sjogren syndrome (*n* = 1), and Sjogren syndrome and dermatomyositis (*n* = 1), followed by sarcoidosis (*n* = 4), scleroderma (*n* = 4), mixed connective tissue disease (*n* = 3), systemic lupus erythematosus (*n* = 1), mixed connective tissue disease and COVID-19 infection (*n* = 1).

### 3.2. Associated Comorbidities and Treatment

In the analysis of associated comorbidities between patients with IPF and SPF, several notable differences were observed, as demonstrated in Table 2. The exacerbation rate was also higher in the IPF group, with 15.79% experiencing exacerbations, whereas no exacerbations were reported in the SPF group (*p*-value = 0.035). Additionally, ischemic heart disease was more prevalent among IPF patients (42.11%) compared to those with SPF (17.86%) (*p*-value = 0.037). There were no significant differences in the presence of chronic obstructive pulmonary disease (COPD), diabetes, and hypertension between the groups. While the use of antifibrotic therapy, specifically Nintedanib, was significantly more common in the IPF group (63.16% vs. 17.86%; *p*-value < 0.001), the use of Pirfenidone did not differ significantly between the groups.

### 3.3. Hepatic Steatosis, Liver Fibrosis, and Hemogram-Derived Ratios

In the comparative analysis of hemogram-derived ratios, hepatic steatosis, and liver fibrosis scores between patients with IPF and SPF, several significant differences were identified, as mentioned in Table 3. The FIB-4 score was significantly higher in the IPF group (median: 1.66) compared to the SPF group (median: 1.42) (*p*-value = 0.049). Additionally, the RDW/PLT was also significantly higher in the IPF group (median: 0.07) compared to the SPF group (median: 0.06) (*p*-value = 0.037). The PLR and PNR were significantly higher in the SPF group with a median of 160.52, compared to 107.24 in the IPF group (*p*-value = 0.005), and a median of 160.52 in the SPF group, compared to 107.24 in the IPF group, with a *p*-value of 0.005. Similarly, the SII was elevated in the SPF group (median: 957.35) versus the IPF group (median: 560.88) (*p*-value = 0.011). The LMR was significantly lower in the SPF group, with a median of 2.52 compared to 3.5 in the IPF group (*p*-value = 0.044).

### 3.4. Pulmonary Function Tests

A comparison of pulmonary function parameters between patients with IPF and SPF is outlined in Table 4, revealing a significant difference in the MEF25%. Specifically, the MEF25% was significantly lower in the SPF group (median: 44) compared to the IPF group (median: 61.5) with a *p*-value of 0.042. However, other pulmonary function metrics, including FVC%, FEV1%, FEV1/FVC, MEF75%, MEF50%, and DLCO, did not show significant differences between the two groups.

### 3.5. Pulmonary Function Tests in Relation to Hemogram-Derived Ratios, Hepatic Steatosis and Liver Fibrosis Scores

#### 3.5.1. Mean Expiratory Flow

As outlined in Supplementary Materials Table S1, the comparison of various biomarkers and scores based on whether the MEF75% was ≥80% revealed the following results. NLR, dNLR, PLR, LMR, SII, PNR, ELR, BLR, FIB-4, APRI, BARD, AST/ALT ratio, ABIC, LogOddsLok, RDW to PLT Ratio, FEV1/FVC, and MEF50% did not show significant differences between the groups with MEF75% ≥ 80% and those with MEF75% < 80%. Nevertheless, King Score was significantly higher in the group with MEF75% < 80% (median: 9.38) compared to the group with MEF75% ≥ 80% (median: 6.67) with a *p*-value of 0.04. Moreover, Lok Index and TyG Ratio showed trends, but did not reach statistical significance, with *p*-values of 0.103 and 0.197, respectively.

The analysis comparing various biomarkers and scores based on whether the MEF50% is ≥80%, is demonstrated in Supplementary Materials Table S2, yielding the following results. PLR was significantly higher in the group with MEF50% < 80% (median: 155.37) compared to the group with MEF50% ≥ 80% (median: 100.84) with a *p*-value of 0.031. SII was significantly higher in the group with MEF50% < 80% (median: 857.27) compared to the group with MEF50% ≥ 80% (median: 576.11) with a *p*-value of 0.045. AST/ALT ratio was significantly higher in the group with MEF50% ≥ 80% (median: 1.44) compared to the group with MEF50% < 80% (median: 1.23) with a *p*-value of 0.042. No significant differences were observed for NLR, dNLR, LMR, PNR, ELR, BLR, FIB-4, APRI, BARD, ABIC, King Score, LogOddsLok, RDW to PLT Ratio, Lok Index, or TyG Ratio between the two groups.

As mentioned in Supplementary Materials Table S3, the analysis of biomarkers and scores in relation to MEF25% being ≥80% revealed the following. ABIC was significantly higher in the group with MEF25% < 80% (median: 8.24) compared to the group with MEF25% ≥ 80% (median: 8.73) with a *p*-value of 0.031. No significant differences were observed for NLR, dNLR, PLR, LMR, SII, PNR, ELR, BLR, FIB-4, APRI, BARD, AST/ALT ratio, King Score, LogOddsLok, RDW to PLT Ratio, Lok Index, or TyG Ratio between the two groups.

#### 3.5.2. DLCO

Supplementary Materials Table S4 summarizes the analysis of biomarkers and scores by DLCO categories, revealing that most indicators, including NLR, dNLR, PLR, LMR, SII, ELR, BLR, FIB-4, APRI, BARD, and AST/ALT ratio, showed no significant differences between mild, moderate, normal, and severe categories. Key measures like the ABIC score, King Score, and RDW-to-PLT Ratio also did not vary significantly. SII and ELR trends were observed, but differences were not statistically significant.

### 3.6. Correlations Between Pulmonary Function Tests and Assessed Biomarkers/Scores

**Figure 1:**
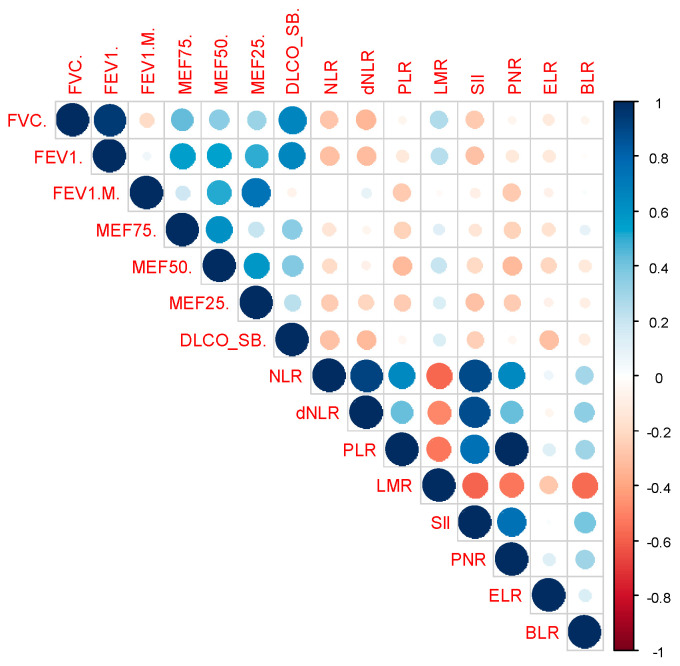
Correlation matrix evaluating pulmonary function tests (FVC%, FEV1%, FEV1/FVC, MEF75%, MEF50%, MEF25%, DLCO) and hemogram-derived ratios (NLR, dNLR, PLR, LMR, SII, PNR, ELR, BLR).

**Figure 2:**
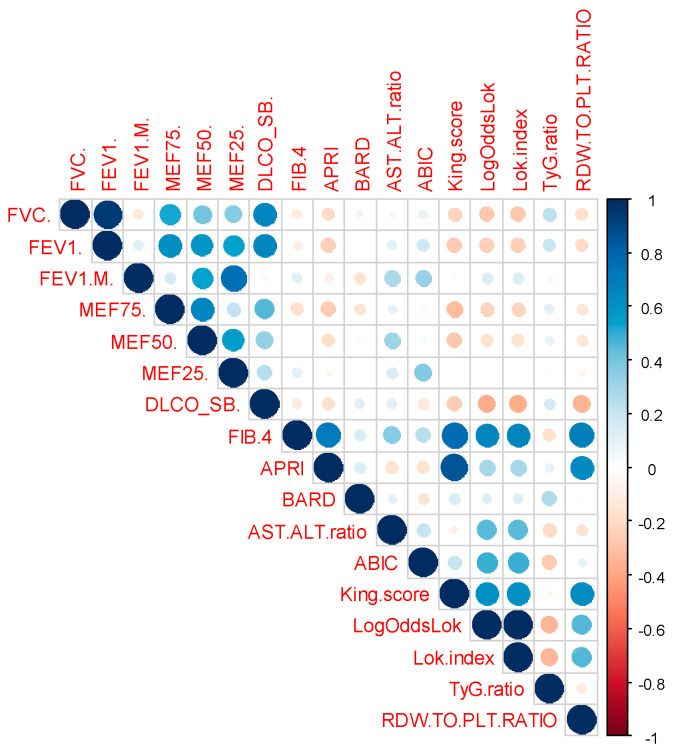
Correlation matrix displaying relationships between pulmonary function tests (FVC%, FEV1%, FEV1/FVC, MEF75%, MEF50%, MEF25%, DLCO), various liver fibrosis scores (FIB-4, APRI, BARD, AST/ALT ratio, ABIC, King score, LogOddsLok, Lok index, TyG ratio), and RDW-to-PLT ratio.

The analysis of correlations between hemogram-derived ratios (Figure 1) and liver fibrosis scores (Figure 2) with lung function parameters revealed several significant findings (Table 5). NLR and dNLR exhibited notable negative correlations with FVC% (*p*-value = 0.022 and *p* = 0.006, respectively) and FEV1% (*p*-value = 0.014 and *p*-value = 0.01, respectively). In contrast, LMR showed a significant positive correlation with FVC% (*p*-value = 0.03). PLR correlates positively with FEV1/FVC (*p*-value = 0.033) and negatively with MEF50% (*p*-value = 0.009). ABIC also shows a significant correlation with FEV1/FVC (*p*-value = 0.026).

For MEF75%, there was a significant negative correlation with LogOddsLok (*p*-value = 0.027). MEF50% was notably negatively correlated with PLR and dNLR (*p*-value = 0.009 for both). MEF25% had significant negative correlations with NLR (*p*-value = 0.038) and PLR (*p*-value = 0.04), along with a positive correlation with King score (*p*-value = 0.005).

DLCO displayed significant negative correlations with NLR (*p*-value = 0.035) and dNLR (*p*-value = 0.012), and a positive correlation with LogOddsLok (*p*-value = 0.049).

Overall, NLR and dNLR were consistently negatively correlated with various lung function measures, while other biomarkers demonstrated less consistent and variable associations.

### 3.7. AUROC to Differentiate Between IPF and SPF

To differentiate between IPF and SPF, various biomarkers were evaluated for their diagnostic performance as outlined in Table 6. Among them, PLR and PNR demonstrated the highest discriminatory ability, with an AUC of 0.702, reflecting good sensitivity (71.43%) and specificity (65.79%). The RDW-to-PLT Ratio also showed strong performance, with an AUC of 0.651 and perfect sensitivity (100%), but it lacked specificity (0%). The SII had an AUC of 0.684, providing high sensitivity (85.71%) and moderate specificity (57.89%). In contrast, FIB-4 and APRI were highly sensitive (100%) but did not effectively differentiate PF, due to their zero specificity. The NLR and dNLR had moderate AUCs (0.623 and 0.604), with balanced sensitivity and specificity. The LMR had high specificity (94.74%) but very low sensitivity (7.14%). Other markers like ABIC and King Score showed lower AUCs (0.591 and 0.642), indicating less reliability. LogOddsLok and Lok Index also had high specificity (96.55%) but very low sensitivity (8.7%). Overall, while FIB-4 and APRI are highly sensitive, PLR and PNR offer a balanced approach, making them more effective for distinguishing pulmonary fibrosis.

### 3.8. Risk of Advanced Hepatic Fibrosis and Pulmonary Function Tests

In assessing advanced hepatic fibrosis risk using FIB-4, spirometric measures showed varying results, as demonstrated in Supplementary Materials Table S5. FVC% and FEV1% did not differ significantly between high, indeterminate, and low fibrosis-risk categories. However, FEV1/FVC was notably higher in the high-risk group. Other measures like MEF75%, MEF50%, MEF25%, and DLCO showed no significant differences across the risk categories.

For advanced fibrosis risk assessed by APRI, spirometric and DLCO measures showed no significant differences between high- and low-risk groups- as reported in Supplementary Materials Table S6. Median values for FVC%, FEV1%, and other spirometric measures like MEF75%, MEF50%, and MEF25% were similar across groups. DLCO also did not differ significantly, indicating limited variability in these parameters relative to APRI-defined fibrosis risk.

When comparing advanced fibrosis risk based on the BARD score, spirometric measures and DLCO did not show significant differences between high- and low-risk groups, as mentioned in Supplementary Materials Table S7. Median values for FVC%, FEV1%, FEV1%M%, MEF75%, MEF50%, MEF25%, and DLCO were similar across both risk categories, with no statistically significant differences found.

### 3.9. Comparative Analysis of Advanced Fibrosis Risk in IPF vs. SPF

In the context of pulmonary fibrosis, IPF cases had a significantly higher proportion of high FIB-4 advanced-fibrosis risk compared to SPF cases (18.42% vs. 0%, *p*-value= 0.015), as outlined in Table 7. APRI advanced-fibrosis risk showed no significant difference between IPF and SPF (5.26% vs. 0%, *p*-value= 0.504). For BARD advanced-fibrosis risk, the proportion of high-risk cases was similar between the two groups (89.47% in IDP vs. 96.43% in SPF, *p*-value = 0.385).

## 4. Discussion

This study investigated the relationship between pulmonary fibrosis, hepatic conditions, and various biomarkers, providing new insights into differentiating IPF from SPF. Our main findings reveal that IPF patients exhibit higher FIB-4 scores and RDW/PLT ratios compared to SPF patients, suggesting increased hepatic fibrosis risk. Conversely, SPF patients show elevated PLR and SII, reflecting a more pronounced inflammatory profile. Notably, PLR and PNR demonstrated the highest discriminatory ability between IPF and SPF, while traditional hepatic fibrosis scores such as FIB-4 and APRI had limited differentiation capabilities. Our analysis also found no significant differences in pulmonary function tests across hepatic fibrosis-risk categories, indicating that hepatic fibrosis risk may not directly impact pulmonary function.

In our study, we observed a significant association between pulmonary fibrosis and hepatic conditions, with IPF patients showing elevated FIB-4 scores and RDW/PLT ratios compared to SPF patients. The FIB-4 score, which combines age, AST, ALT, and platelet count, is a validated non-invasive marker of hepatic fibrosis. Higher FIB-4 scores in IPF align with findings from Cocconcelli et al., who reported a notable overall survival risk related to liver fibrosis risk in IPF patients [15]. This association may be attributed to shared pathogenic mechanisms such as chronic inflammation and oxidative stress, which drive both pulmonary and hepatic fibrosis.

Conversely, SPF patients exhibited higher PLR and SII. Elevated PLR and SII in SPF could reflect the inflammatory and immune dysregulation often observed in secondary causes of pulmonary fibrosis, such as autoimmune diseases or occupational exposures. The heightened inflammatory profile in SPF may contribute to distinct clinical and histopathological features compared to IPF. Our analysis identified PLR and PNR as particularly effective in differentiating between IPF and SPF, with PLR showing the highest discriminatory power among the evaluated biomarkers. PLR has been increasingly recognized for its role in various inflammatory and fibrotic diseases. Achaiah et al. demonstrated that blood neutrophil and lymphocyte counts are more reliable than monocytes for predicting disease progression in individuals with established IPF [25]. Chen et al. found that higher levels of NLR expression correlate with reduced overall survival in patients with IPF, regardless of other prognostic factors. This suggests that NLR could serve as a dependable prognostic biomarker for individuals with IPF [26].

However, FIB-4 and APRI, despite their high sensitivity, did not effectively distinguish between IPF and SPF. The discrepancy may stem from differences in patient populations or the specific characteristics of pulmonary fibrosis. Our findings suggest that while these scores are useful for assessing hepatic fibrosis, they may not fully capture the complexities of pulmonary fibrosis. The lack of significant differences in pulmonary function tests across hepatic fibrosis-risk categories (assessed by FIB-4, APRI, and BARD) is noteworthy. Our results suggest that while hepatic fibrosis risk may influence lung function, the relationship might be less direct or more complex than previously thought [27]. Factors such as concurrent comorbidities, disease duration, and treatment effects could contribute to these nuanced findings. Our results indicate a higher proportion of advanced fibrosis risk (as assessed by FIB-4) in IPF compared to SPF, corroborating earlier studies, which found a strong link between IPF and liver fibrosis. The similar proportions of high-risk cases for APRI and BARD between IPF and SPF suggest that these scores may not be as effective in distinguishing between the two conditions. This could be due to the overlap in the underlying mechanisms of fibrosis and the influence of various confounding factors.

Our study underscores the utility of biomarkers such as the PLR and PNR in differentiating IPF from SPF. These biomarkers offer a valuable complement to traditional diagnostic methods, which primarily rely on HRCT and sometimes lung biopsy. While HRCT is effective in identifying the UIP pattern, it may not always clearly distinguish between IPF and SPF, especially in complex cases [28]. PLR and PNR reflect systemic inflammation and immune responses, providing additional diagnostic insights that can enhance accuracy. Integrating these biomarkers into clinical practice can improve diagnostic precision and patient management by offering a quantitative measure of inflammation that supports a more comprehensive and nuanced approach to distinguishing between IPF and SPF.

It is important to mention that antifibrotic medications, including Nintedanib and Pirfenidone, play a critical role in managing IPF by slowing disease progression and improving patient outcomes. However, these medications can pose risks of hepatotoxicity [29]. Nintedanib, a tyrosine kinase inhibitor, has been associated with mild and generally reversible elevations in liver enzymes, necessitating regular monitoring of liver function throughout treatment [30]. Similarly, Pirfenidone, an anti-inflammatory and antifibrotic agent, can cause increases in transaminases and bilirubin levels, though it is usually well-tolerated when managed appropriately [31]. Both drugs have been linked to cases of DILI, underscoring the importance of close surveillance for hepatic adverse effects. The risk of hepatotoxicity may require dose adjustments or discontinuation of therapy, highlighting the need to carefully balance the therapeutic benefits of these medications against potential liver-related risks. Effective management of these hepatic side effects is essential for optimizing patient care and minimizing severe liver complications associated with IPF treatment [5].

Our findings underscore the importance of integrating hepatic assessments into the management of pulmonary fibrosis. Given the high prevalence of hepatic involvement in IPF and the distinct inflammatory profiles in SPF, clinicians should consider a holistic approach to evaluating patients with pulmonary fibrosis. The use of biomarkers such as PLR and PNR can enhance diagnostic accuracy and guide personalized treatment strategies. Future research should focus on validating these findings in larger, multi-center cohorts and exploring the underlying mechanisms linking hepatic and pulmonary fibrosis. Longitudinal studies assessing the impact of hepatic involvement on disease progression and treatment response in pulmonary fibrosis are also warranted. Such research will help refine diagnostic tools and therapeutic approaches, ultimately improving patient outcomes in this challenging field.

Our study has several limitations that should be considered when interpreting the results. First, its retrospective design may introduce selection bias, as it relies on existing patient records and clinical data, which can limit the generalizability of our findings. Additionally, the sample size, while adequate for preliminary analyses, may not be large enough to detect more subtle differences between IPF and SPF. The lack of a longitudinal follow-up means that we could not assess the long-term outcomes or progression of disease in relation to the biomarkers studied. Furthermore, although we evaluated various biomarkers and hepatic fibrosis scores, the absence of a gold standard for diagnosing hepatic fibrosis limits our ability to definitively validate the efficacy of these measures. Lastly, the study excluded patients with certain comorbid conditions and incomplete data, which might have affected the representativeness of the patient cohort.

Despite these limitations, our study presents several strengths that enhance its contribution to the field. The inclusion of a comprehensive set of biomarkers and hepatic fibrosis scores provides a robust analysis of their potential utility in differentiating between IPF and SPF. By incorporating both traditional diagnostic methods and novel biomarkers, the study offers a holistic approach to understanding pulmonary fibrosis and its associated hepatic involvement. The use of well-defined criteria for diagnosing IPF and SPF, based on HRCT and clinical evaluation, adds to the accuracy of our classifications. Additionally, the significant findings regarding the utility of PLR and PNR in distinguishing between IPF and SPF provide valuable insights for clinical practice and suggest directions for future research. Overall, the study’s thorough examination of these biomarkers contributes valuable information to the ongoing efforts to refine diagnostic and prognostic tools for pulmonary fibrosis.

## 5. Conclusions

IPF patients have higher FIB-4 scores, indicating greater hepatic fibrosis risk, and RDW/PLT ratio, while SPF patients exhibit higher PLR and SII, reflecting a stronger inflammatory response. PLR and PNR were the most effective at distinguishing IPF from SPF, whereas traditional fibrosis scores like FIB-4 and APRI were less discriminative. Additionally, hepatic fibrosis risk did not significantly affect pulmonary function test results.

## Figures and Tables

**Table 1:** General characteristics and laboratory tests of included participants.

Variables	IPF (*n* = 38)	SPF (*n* = 28)	*p*-Value
Age (years), median (IQR)	73 (67.5–75.75)	68 (62.25–74)	0.021
Sex (Male), nr (%)	23 (60.53)	9 (32.14)	0.023
BMI (kg/m^2^), median (IQR)	27.87 (25.59–30.31)	28.65 (26.31–29.96)	0.825
BMI interpretation, nr (%)	Normal: 6 (15.79)	Normal: 6 (21.43)	0.841
	Obesity: 10 (26.32)	Obesity: 7 (25)	
	Overweight: 22 (57.89)	Overweight: 15 (53.57)	
Smoking history, nr (%)	Ex-smoker: 15 (39.47)	Ex-smoker: 7 (25)	0.363
	Non-smoker: 21 (55.26)	Non-smoker: 18 (64.29)	
	Smoker: 2 (5.26)	Smoker: 3 (10.71)	
Smoking (pack/years), median (IQR)	20 (17–29)	30 (20–30)	0.45
Professional exposure (Yes), nr (%)	10 (26.32)	8 (28.57)	0.839
Atopic risk (Yes), nr (%)	5 (13.16)	8 (28.57)	0.12
CRP (mg/L), median (IQR)	6 (1.25–22.67)	7.5 (3–16.62)	0.617
AST (UI/L), median (IQR)	22.5 (19–27.25)	22.5 (17.75–27.5)	0.697
ALT (UI/L), median (IQR)	17.5 (13.25–26)	16 (11–20.5)	0.32
Total bilirubin (mg/dL), median (IQR)	0.74 (0.46–1.11)	0.52 (0.31–0.71)	0.011
ALP (UI/L), median (IQR)	97.5 (69.25–137.75)	85 (69.5–103.5)	0.392
GGT (UI/L), median (IQR)	29.5 (17.25–50.5)	22 (14.5–30.25)	0.099
INR, median (IQR)	1.12 (1.04–1.24)	1.08 (1.04–1.27)	0.455
PT (s), median (IQR)	12.7 (11.4–14)	12 (11.55–13.9)	0.632
Urea (mg/dL), median (IQR)	37 (28.5–45.5)	32 (25–42.5)	0.192
Creatinine (mg/dL), median (IQR)	0.9 (0.72–1.04)	0.81 (0.68–1)	0.452
eGFR, mean (SD)	76.9 (21.85)	77.42 (25.02)	0.929
Total cholesterol (mg/dL), mean (SD)	168.43 (48.95)	175.54 (44.57)	0.547
Triglycerides (mg/dL), median (IQR)	113.5 (84–149.5)	120 (94.5–164.5)	0.523
RDW-CV (%), median (IQR)	13.8 (12.83–14.67)	14.45 (13.28–15.33)	0.109

ALP: Alkaline Phosphatase; ALT: Alanine Aminotransferase; AST: Aspartate Aminotransferase; BMI: Body Mass Index; CRP: C-reactive Protein; eGFR: Estimated Glomerular Filtration Rate; GGT: Gamma-Glutamyl Transferase; INR: International Normalized Ratio; IPF: Idiopathic Pulmonary Fibrosis; IQR: Interquartile Range; PT: Prothrombin Time; RDW-CV: Red Cell Distribution Width–Coefficient of Variation; SD: Standard Deviation; SPF: Secondary Pulmonary Fibrosis; UI/L: Units per Liter.

**Table 2:** Associated comorbidities and administered treatment in IPF and SPF.

Variables	IPF (*n* = 38)	SPF (*n* = 28)	*p*-Value
Comorbidities			
Exacerbation (Yes), nr (%)	6 (15.79)	0 (0)	0.035
COPD (Yes), nr (%)	5 (13.16)	5 (17.86)	0.732
COPD exacerbation (Yes), nr (%)	1 (2.63)	1 (3.57)	1
Asthma (Yes), nr (%)	4 (10.53)	4 (14.29)	0.714
Bronchiectasis (Yes), nr (%)	20 (52.63)	14 (50)	0.833
Home oxygen therapy (Yes), nr (%)	18 (47.37)	7 (25)	0.064
Obstructive sleep apnea (Yes), nr (%)	1 (2.63)	2 (7.14)	0.57
COVID-19 (Yes), nr (%)	8 (21.05)	7 (25)	0.705
Diabetes (Yes), nr (%)	11 (28.95)	5 (17.86)	0.299
Hypertension (Yes), nr (%)	29 (76.32)	18 (64.29)	0.286
Heart failure (Yes), nr (%)	14 (36.84)	10 (35.71)	0.925
Ischemic heart disease (Yes), nr (%)	16 (42.11)	5 (17.86)	0.037
Previous MI (Yes), nr (%)	2 (5.26)	3 (10.71)	0.643
Pulmonary hypertension (Yes), nr (%)	21 (55.26)	13 (46.43)	0.478
Cerebrovascular accident (Yes), nr (%)	0 (0)	2 (7.14)	0.176
Carotid atherosclerosis (Yes), nr (%)	7 (18.42)	7 (25)	0.518
Treatment			
Corticosteroids (Yes), nr (%)	2 (5.26)	3 (10.71)	0.643
Biological treatment (Yes), nr (%)	0 (0)	2 (7.14)	0.176
Biological treatment type (RITUXIMAB), nr (%)	0 (0)	2 (7.14)	0.176
Nintedanib, nr (%)	Initiated: 6 (15.79)	Initiated: 2 (7.14)	<0.001
	Interrupted: 3 (7.89)	Interrupted: 0 (0)	
	No: 5 (13.16)	No: 21 (75)	
	Yes: 24 (63.16)	Yes: 5 (17.86)	
Pirfenidone, nr (%)	Initiated: 1 (2.63)	Initiated: 0 (0)	0.256
	No: 34 (89.47)	No: 28 (100)	
	Yes: 3 (7.89)	Yes: 0 (0)	

COPD: Chronic Obstructive Pulmonary Disease; COVID-19: Coronavirus disease; IPF: Idiopathic Pulmonary Fibrosis; MI: Myocardial Infarction; SPF: Secondary Pulmonary Fibrosis.

**Table 3:** Evaluated hemogram-derived ratios, hepatic steatosis and liver fibrosis scores.

Variables	IPF (*n* = 38)	SPF (*n* = 28)	*p*-Value
Hemogram-derived ratios			
NLR, median (IQR)	2.75 (2.09–4.06)	3.65 (2.57–4.99)	0.091
dNLR, median (IQR)	14.16 (7.92–25.46)	19.07 (12.37–33.89)	0.153
PLR, median (IQR)	107.24 (78.59–163.32)	160.52 (119.64–265.17)	0.005
LMR, median (IQR)	3.5 (2.54–5.05)	2.52 (1.93–4)	0.044
SII, median (IQR)	560.88 (386.72–999.12)	957.35 (669.49–1308.34)	0.011
PNR, median (IQR)	107.24 (78.59–163.32)	160.52 (119.64–265.17)	0.005
ELR, median (IQR)	0.12 (0.05–0.2)	0.11 (0.08–0.18)	0.756
BLR, median (IQR)	0.01 (0–0.03)	0.02 (0.01–0.03)	0.081
RDW TO PLT RATIO, median (IQR)	0.07 (0.05–0.08)	0.06 (0.04–0.07)	0.037
Hepatic steatosis and liver fibrosis scores			
FIB-4, median (IQR)	1.66 (1.35–2.23)	1.42 (1.07–1.98)	0.049
APRI, median (IQR)	0.33 (0.2–0.43)	0.23 (0.17–0.35)	0.05
BARD, median (IQR)	3 (2–3)	3 (2–3)	0.55
AST/ALT ratio, median (IQR)	1.27 (0.95–1.57)	1.39 (1.16–1.64)	0.246
ABIC, median (IQR)	8.67 (8.19–8.76)	8.25 (7.33–8.76)	0.272
King score, median (IQR)	9.34 (6.34–11.34)	7.02 (4.91–9.9)	0.083
LogOddsLok, median (IQR)	0.29 (−0.74–1)	−0.46 (−1.22–0.3)	0.101
Lok index, mean (SD)	0.55 (0.25)	0.43 (0.26)	0.117
TyG ratio, mean (SD)	3.78 (0.21)	3.8 (0.27)	0.793

ALT: Alanine Aminotransferase; AST: Aspartate Aminotransferase; ABIC: Age-Bilirubin-INR-Creatinine Score; APRI: Aspartate Aminotransferase-to-Platelet Ratio Index; BARD: Bilirubin, Age, AST/ALT Ratio, and Diabetes Score; BLR: Basophil-to-Lymphocyte Ratio; ELR: Eosinophil-to-Lymphocyte Ratio; FIB-4: Fibrosis-4 Index; IPF: Idiopathic Pulmonary Fibrosis; IQR: Interquartile Range; King score: King’s Score for Liver Fibrosis; LMR: Lymphocyte-to-Monocyte Ratio; LogOddsLok: Log Odds of Lok Index; NLR: Neutrophil-to-Lymphocyte Ratio; PLR: Platelet-to-Lymphocyte Ratio; PNR: Platelet-to-Neutrophil Ratio; RDW TO PLT RATIO: Red Cell Distribution Width-to-Platelet Ratio; SII: Systemic Immune–Inflammation Index; SD: Standard Deviation; SPF: Secondary Pulmonary Fibrosis; TyG ratio: Triglyceride Glucose Index.

**Table 4:** Pulmonary function tests according to pulmonary fibrosis type.

Variables	IPF (*n* = 38)	SPF (*n* = 28)	*p*-Value
FVC%, mean (SD)	76.08 (23.21)	76.11 (25)	0.996
FEV1%, mean (SD)	79.47 (22.33)	76.81 (27.09)	0.667
FEV1/FVC, median (IQR)	109 (105–114)	105 (98.5–111)	0.164
MEF75%, mean (SD)	78.24 (28.19)	83.33 (33.44)	0.509
MEF50%, median (IQR)	78.5 (58–93.5)	68 (46.5–92)	0.405
MEF25%, median (IQR)	61.5 (48–82.5)	44 (27–71.5)	0.042
MEF75% ≥ 80% (Yes), nr (%)	16 (42.11)	16 (59.26)	0.173
MEF50% ≥ 80% (Yes), nr (%)	19 (50)	12 (44.44)	0.659
MEF25% ≥ 80% (Yes), nr (%)	11 (28.95)	6 (22.22)	0.543
DLCO, mean (SD)	46.61 (16.96)	56.5 (26.77)	0.093
DLCO category, nr (%)	Mild: 10 (26.32)	Mild: 7 (25)	0.466
	Moderate: 12 (31.58)	Moderate: 8 (28.57)	
	Normal: 2 (5.26)	Normal: 5 (17.86)	
	Severe: 14 (36.84)	Severe: 8 (28.57)	

DLCO: Diffusing Capacity for Carbon Monoxide; FEV1%: Forced Expiratory Volume in 1 s percentage; FEV1/FVC: Ratio of FEV1 to FVC; FVC%: Forced Vital Capacity percentage; IPF: Idiopathic Pulmonary Fibrosis; IQR: Interquartile Range; MEF25%: Mid-Expiratory Flow at 25% of FVC; MEF50%: Mid-Expiratory Flow at 50% of FVC; MEF75%: Mid-Expiratory Flow at 75% of FVC; SD: Standard Deviation; SPF: Secondary Pulmonary Fibrosis.

**Table 5:** The association between hemogram-derived ratios, hepatic steatosis, and liver fibrosis scores with pulmonary function tests.

Variable	NLR	dNLR	PLR	LMR	SII	PNR	ELR	BLR	RDW to PLT Ratio	FIB-4	APRI	BARD	AST/ALT Ratio	ABIC	King Score	LogOddsLok	Lok Index	TyG Ratio
FVC%	−0.28 (0.022)	−0.34 (0.006)	−0.05 (0.68)	0.27 (0.03)	−0.26 (0.033)	−0.05 (0.68)	−0.12 (0.358)	−0.06 (0.641)	−0.02 (0.888)	0.05 (0.689)	−0.02 (0.844)	0.02 (0.863)	−0.05 (0.698)	0.14 (0.343)	−0.16 (0.272)	−0.2 (0.161)	−0.2 (0.161)	0.15 (0.233)
FEV1%	−0.3 (0.014)	−0.32 (0.01)	−0.13 (0.305)	0.24 (0.053)	−0.3 (0.017)	−0.13 (0.305)	−0.13 (0.308)	−0.02 (0.901)	−0.01 (0.93)	0.08 (0.525)	−0.04 (0.742)	−0.02 (0.88)	0.02 (0.864)	0.22 (0.129)	−0.2 (0.158)	−0.18 (0.205)	−0.18 (0.205)	0.11 (0.382)
FEV1/FVC	−0.01 (0.966)	0.08 (0.526)	−0.26 (0.033)	−0.02 (0.857)	−0.09 (0.494)	−0.26 (0.033)	−0.07 (0.564)	0.01 (0.912)	−0.01 (0.965)	0.1 (0.441)	−0.02 (0.851)	−0.11 (0.38)	0.28 (0.026)	0.3 (0.032)	−0.03 (0.828)	0.11 (0.431)	0.11 (0.431)	−0.09 (0.47)
MEF75%	−0.15 (0.235)	−0.06 (0.653)	−0.24 (0.058)	0.11 (0.376)	−0.14 (0.252)	−0.24 (0.058)	−0.16 (0.198)	0.1 (0.448)	−0.11 (0.388)	−0.08 (0.531)	−0.13 (0.291)	−0.1 (0.434)	0.08 (0.55)	−0.03 (0.831)	−0.31 (0.027)	−0.21 (0.131)	−0.21 (0.131)	0.1 (0.421)
MEF50%	−0.2 (0.116)	−0.08 (0.549)	−0.32 (0.009)	0.2 (0.106)	−0.21 (0.095)	−0.32 (0.009)	−0.22 (0.085)	−0.12 (0.323)	−0.07 (0.554)	0.04 (0.769)	−0.06 (0.645)	0 (0.994)	0.18 (0.141)	0.08 (0.579)	−0.23 (0.11)	−0.12 (0.396)	−0.12 (0.396)	0.07 (0.583)
MEF25%	−0.26 (0.038)	−0.21 (0.087)	−0.26 (0.04)	0.13 (0.284)	−0.3 (0.017)	−0.26 (0.04)	−0.07 (0.558)	−0.09 (0.467)	0.01 (0.919)	0.12 (0.357)	0.03 (0.784)	−0.04 (0.749)	0.11 (0.381)	0.39 (0.005)	0.04 (0.8)	0.03 (0.814)	0.03 (0.814)	−0.09 (0.486)
DLCO	−0.26 (0.035)	−0.31 (0.012)	−0.02 (0.875)	0.11 (0.399)	−0.21 (0.087)	−0.02 (0.875)	−0.26 (0.033)	−0.13 (0.297)	−0.19 (0.118)	−0.01 (0.912)	−0.06 (0.639)	0.09 (0.47)	0.01 (0.917)	−0.03 (0.858)	−0.17 (0.234)	−0.27 (0.049)	−0.27 (0.049)	0.17 (0.188)

ABIC: Age-Bilirubin-INR-Creatinine Score; APRI: AST-to-Platelet Ratio Index; AST/ALT Ratio: Aspartate Aminotransferase-to-Alanine Aminotransferase Ratio; BARD: BMI, AST/ALT Ratio, Diabetes Score; BLR: Basophil-to-Lymphocyte Ratio; DLCO: Diffusing Capacity of the Lungs for Carbon Monoxide; dNLR: Derived Neutrophil-to-Lymphocyte Ratio; ELR: Eosinophil-to-Lymphocyte Ratio; FEV1%: Forced Expiratory Volume in One-Second Percent Predicted; FEV1/FVC: Ratio of FEV1 to FVC; FIB-4: Fibrosis-4 Index; FVC%: Forced Vital Capacity Percent Predicted; LMR: Lymphocyte-to-Monocyte Ratio; Lok index: Lok Index; LogOddsLok: Log Odds of Lok Model; MEF25%: Maximal Expiratory Flow at 25% of FVC; MEF50%: Maximal Expiratory Flow at 50% of FVC; MEF75%: Maximal Expiratory Flow at 75% of FVC; NLR: Neutrophil-to-Lymphocyte Ratio; PNR: Platelet-to-Neutrophil Ratio; PLR: Platelet-to-Lymphocyte Ratio; RDW-to-PLT Ratio: Red Cell Distribution-Width-to-Platelet Ratio; SII: Systemic Immune–Inflammation Index; TyG Ratio: Triglyceride Glucose Index Ratio.

**Table 6:** AUROC of several hemogram-derived ratios, hepatic steatosis, and liver fibrosis scores for differentiating IPF from SPF.

Variable	AUC (95% CI)	Se	Sp	Cut-Off
RDW-CV (%)	0.617 (0.48–0.75)	50	71.05	14.4
NLR	0.623 (0.481–0.758)	60.71	65.79	3.283261803
dNLR	0.604 (0.461–0.743)	67.86	60.53	17.36452174
PLR	0.702 (0.569–0.824)	71.43	65.79	135.042735
LMR	0.646 (0.507–0.784)	7.14	94.74	7.114285714
SII	0.684 (0.548–0.812)	85.71	57.89	607.1794872
PNR	0.702 (0.565–0.827)	71.43	65.79	135.042735
ELR	0.477 (0.338–0.619)	78.57	36.84	0.070422535
BLR	0.626 (0.483–0.761)	85.71	39.47	0.00862
FIB-4	0.643 (0.496–0.773)	100	0	−Inf
APRI	0.642 (0.508–0.776)	100	0	−Inf
BARD	0.541 (0.403–0.672)	100	7.89	0
AST/ALT ratio	0.585 (0.451–0.723)	60.71	57.89	1.285714286

APRI: AST-to-Platelet Ratio Index; ALT: Alanine Aminotransferase; AST: Aspartate Aminotransferase; AUROC: Area Under the Receiver Operating Characteristic Curve; BARD: Bilirubin, Age, AST/ALT Ratio, and Diabetes Score; BLR: Basophil-to-Lymphocyte Ratio; dNLR: Derived Neutrophil-to-Lymphocyte Ratio; ELR: Eosinophil-to-Lymphocyte Ratio; FIB-4: Fibrosis-4 Index; LMR: Lymphocyte-to-Monocyte Ratio; NLR: Neutrophil-to-Lymphocyte Ratio; PLR: Platelet-to-Lymphocyte Ratio; PNR: Platelet-to-Neutrophil Ratio; RDW-CV: Red Cell Distribution-Width–Coefficient of Variation; SII: Systemic Immune–Inflammation Index; Se: Sensitivity; Sp: Specificity.

**Table 7:** Type of pulmonary fibrosis and associated risk of advanced liver fibrosis assessed using FIB-4, APRI, and BARD.

Variable	IPF (*n* = 38)	SPF (*n* = 28)	*p*-Value
FIB-4 Advanced-fibrosis risk, nr (%)	High: 7 (18.42)	High: 0 (0)	0.015
	Indeterminate: 23 (60.53)	Indeterminate: 16 (57.14)	
	Low: 8 (21.05)	Low: 12 (42.86)	
APRI Advanced-fibrosis risk (High), nr (%)	2 (5.26)	0 (0)	0.504
BARD Advanced-fibrosis risk (High), nr (%)	34 (89.47)	27 (96.43)	0.385

APRI: AST-to-Platelet Ratio Index; BARD: Bilirubin, Age, AST/ALT Ratio, and Diabetes Score; FIB-4: Fibrosis-4 Index; IPF: Idiopathic Pulmonary Fibrosis; SPF: Secondary Pulmonary Fibrosis.

## Data Availability

The datasets from the current study can be made available by the corresponding author upon reasonable request.

## Supplementary Materials

The following supporting information can be downloaded at https://www.mdpi.com/article/10.3390/medicina60101702/s1, Table S1: Hemogram-derived ratios, hepatic steatosis, and liver fibrosis scores according to MEF75% ≥ 80%; Table S2. Hemogram-derived ratios, hepatic steatosis, and liver fibrosis scores according to MEF50% ≥ 80%; Table S3. Hemogram-derived ratios, hepatic steatosis, and liver fibrosis scores according to MEF25% ≥ 80%; Table S4. Hemogram-derived ratios, hepatic steatosis, and liver fibrosis scores according to DLCO categories (Normal: >75% of predicted, up to 140%; Mild: 60–75%; Moderate: 40–60%; Severe: <40%); Table S5. The association between pulmonary function tests and risk of advanced liver fibrosis assessed using FIB-4 score; Table S6. The association between pulmonary function tests and risk of advanced liver fibrosis assessed using APRI score; Table S7. The association between pulmonary function tests and risk of advanced liver fibrosis assessed using BARD score.
