# Supplementary material for: Exploring the Role of Hemogram-Derived Ratios and Liver Fibrosis Scores in Pulmonary Fibrosis
Source: Medicina (Kaunas). 2024 Oct 16;60(10):1702. doi: 10.3390/medicina60101702 (PMC11509704; doi:10.3390/medicina60101702)
Supplement: Supplementary file 1 [file medicina-60-01702-s001.zip › medicina-3199745-supplementary.pdf]

Supplementary Table S1. Hemogram-derived ratios, hepatic steatosis, and liver fibrosis scores according to MEF75% ≥ 80%

**Variable**	**MEF75% < 80% (*n*=33)**	**MEF75% ≥ 80% (*n*=32)**	***p*-value**
NLR, median (IQR)	3.38 (2.57 - 4.13)	2.65 (1.97 - 4.69)	0.364
dNLR, median (IQR)	17.5 (9.92 - 33.31)	16.14 (9.04 - 27.01)	0.83
PLR, median (IQR)	146.41 (96.8 - 188.46)	107.04 (83.27 - 203.86)	0.415
LMR, median (IQR)	2.86 (2.23 - 4.8)	3.29 (2.09 - 4.66)	0.644
SII, median (IQR)	779.46 (507.76 - 1149.07)	718.1 (445.86 - 1062.4)	0.607
PNR, median (IQR)	146.41 (96.8 - 188.46)	107.04 (83.27 - 203.86)	0.415
ELR, median (IQR)	0.12 (0.06 - 0.2)	0.09 (0.06 - 0.18)	0.555
BLR, median (IQR)	0.01 (0 - 0.03)	0.01 (0.01 - 0.03)	0.332
FIB-4, median (IQR)	1.52 (1.19 - 2.23)	1.48 (1.23 - 1.82)	0.485
APRI, median (IQR)	0.3 (0.19 - 0.44)	0.25 (0.19 - 0.36)	0.319
BARD, median (IQR)	3 (2 - 3)	2.5 (2 - 3)	0.779
AST/ALT ratio, median (IQR)	1.28 (0.92 - 1.59)	1.31 (1.15 - 1.59)	0.479
ABIC, median (IQR)	8.38 (7.76 - 8.75)	8.35 (7.52 - 8.76)	0.801
King score, median (IQR)	9.38 (6.48 - 12.53)	6.67 (5.49 - 9.36)	0.04
LogOddsLok, median (IQR)	0.26 (-0.73 - 1.2)	-0.46 (-0.98 - 0.34)	0.139
RDW TO PLT RATIO, median (IQR)	0.07 (0.05 - 0.07)	0.06 (0.04 - 0.07)	0.27
Lok index, mean (SD)	0.56 (0.29)	0.44 (0.23)	0.103
TyG ratio, mean (SD)	3.75 (0.24)	3.83 (0.24)	0.197

ABIC: Age-Bilirubin-INR-Creatinine Score; APRI: Aspartate Aminotransferase to Platelet Ratio Index; AST/ALT ratio: Aspartate Aminotransferase/Alanine Aminotransferase Ratio; BARD: Body mass index, AST/ALT ratio, Diabetes; BLR: Basophil-to-Lymphocyte Ratio; dNLR: Derived Neutrophil-to-Lymphocyte Ratio; ELR: Eosinophil-to-Lymphocyte Ratio; FIB-4: Fibrosis-4 Index; King score: King's College Hospital Score; LMR: Lymphocyte-to-Monocyte Ratio; Lok index: Lok Fibrosis Index; LogOddsLok: Log-Odds of Lok Index; MEF75%: Maximum Expiratory Flow at 75% of Forced Vital Capacity; NLR: Neutrophil-to-Lymphocyte Ratio; PLR: Platelet-to-Lymphocyte Ratio; PNR: Platelet-to-Neutrophil Ratio; RDW to PLT Ratio: Red Cell Distribution Width to Platelet Ratio; SII: Systemic Immune-Inflammation Index; TyG ratio: Triglyceride-Glucose Index.

Supplementary Table S2. Hemogram-derived ratios, hepatic steatosis, and liver fibrosis scores according to MEF50% ≥ 80%

**Variable**	**MEF50% < 80% (*n*=34)**	**MEF50% ≥ 80% (*n*=31)**	***p*-value**
NLR, median (IQR)	3.33 (2.58 - 4.1)	2.37 (1.79 - 4.38)	0.114
dNLR, median (IQR)	17.55 (10.7 - 31.67)	14.4 (7.15 - 30.43)	0.311
PLR, median (IQR)	155.37 (105.2 - 210.29)	100.84 (78.96 - 159.43)	0.031
LMR, median (IQR)	2.76 (1.99 - 4.24)	3.67 (2.29 - 4.98)	0.144
SII, median (IQR)	857.27 (605.52 - 1162.54)	576.11 (376.59 - 957.35)	0.045
PNR, median (IQR)	155.37 (105.2 - 210.29)	100.84 (78.96 - 159.43)	0.031
ELR, median (IQR)	0.15 (0.06 - 0.2)	0.09 (0.06 - 0.13)	0.174
BLR, median (IQR)	0.02 (0.01 - 0.03)	0.01 (0.01 - 0.02)	0.18
FIB-4, median (IQR)	1.45 (1.15 - 2.07)	1.57 (1.31 - 2.09)	0.357
APRI, median (IQR)	0.26 (0.19 - 0.42)	0.29 (0.19 - 0.39)	0.875
BARD, median (IQR)	3 (2 - 3)	3 (2 - 3)	0.888
AST/ALT ratio, median (IQR)	1.23 (0.93 - 1.44)	1.44 (1.19 - 1.78)	0.042
ABIC, median (IQR)	8.25 (7.7 - 8.77)	8.67 (7.9 - 8.75)	0.757
King score, median (IQR)	8.01 (5.17 - 11.82)	7.42 (5.98 - 9.62)	0.435
LogOddsLok, median (IQR)	0.04 (-0.97 - 1.01)	-0.25 (-0.86 - 0.34)	0.714
RDW TO PLT RATIO, median (IQR)	0.06 (0.05 - 0.07)	0.06 (0.04 - 0.07)	0.641
Lok index, mean (SD)	0.51 (0.29)	0.48 (0.23)	0.699
TyG ratio, mean (SD)	3.78 (0.22)	3.8 (0.25)	0.794

ABIC: Age-Bilirubin-INR-Creatinine Score; APRI: Aspartate Aminotransferase to Platelet Ratio Index; AST/ALT ratio: Aspartate Aminotransferase/Alanine Aminotransferase Ratio; BARD: Body mass index, AST/ALT ratio, Diabetes; BLR: Basophil-to-Lymphocyte Ratio; dNLR: Derived Neutrophil-to-Lymphocyte Ratio; ELR: Eosinophil-to-Lymphocyte Ratio; FIB-4: Fibrosis-4 Index; King score: King's College Hospital Score; LMR: Lymphocyte-to-Monocyte Ratio; Lok index: Lok Fibrosis Index; LogOddsLok: Log-Odds of Lok Index; MEF50%: Maximum Expiratory Flow at 50% of Forced Vital Capacity; NLR: Neutrophil-to-Lymphocyte Ratio; PLR: Platelet-to-Lymphocyte Ratio; PNR: Platelet-to-Neutrophil Ratio; RDW to PLT Ratio: Red Cell Distribution Width to Platelet Ratio; SII: Systemic Immune-Inflammation Index; TyG ratio: Triglyceride-Glucose Index.

Supplementary Table S3. Hemogram-derived ratios, hepatic steatosis, and liver fibrosis scores according to MEF25% ≥ 80%

**Variable**	**MEF25% < 80% (*n*=48)**	**MEF25% ≥ 80% (*n*=17)**	***p*-value**
NLR, median (IQR)	3.25 (2.35 - 4.64)	2.29 (1.82 - 3.86)	0.09
dNLR, median (IQR)	17.75 (10.78 - 33.14)	11.59 (5.25 - 27.77)	0.209
PLR, median (IQR)	141.92 (97.51 - 212.36)	94.87 (71.81 - 160.32)	0.069
LMR, median (IQR)	3.21 (2.04 - 4.83)	2.79 (2.56 - 4.66)	0.959
SII, median (IQR)	800.05 (503.79 - 1212.4)	604.96 (320.37 - 923.73)	0.069
PNR, median (IQR)	141.92 (97.51 - 212.36)	94.87 (71.81 - 160.32)	0.069
ELR, median (IQR)	0.11 (0.04 - 0.21)	0.1 (0.07 - 0.15)	0.743
BLR, median (IQR)	0.02 (0 - 0.03)	0.01 (0.01 - 0.03)	0.799
FIB-4, median (IQR)	1.5 (1.21 - 2)	1.58 (1.19 - 2.19)	0.641
APRI, median (IQR)	0.27 (0.19 - 0.43)	0.27 (0.2 - 0.34)	0.726
BARD, median (IQR)	3 (2 - 3)	3 (2 - 3)	0.867
AST/ALT ratio, median (IQR)	1.27 (0.96 - 1.59)	1.4 (1.17 - 1.57)	0.31
ABIC, median (IQR)	8.24 (7.34 - 8.71)	8.73 (8.31 - 8.76)	0.031
King score, median (IQR)	8.24 (5.8 - 11.34)	6.56 (5.94 - 9.3)	0.397
LogOddsLok, median (IQR)	-0.28 (-0.91 - 1.04)	0.14 (-0.75 - 0.39)	0.669
RDW TO PLT RATIO, median (IQR)	0.06 (0.05 - 0.07)	0.06 (0.05 - 0.07)	0.946
Lok index, mean (SD)	0.51 (0.29)	0.46 (0.18)	0.440
TyG ratio, mean (SD)	3.8 (0.22)	3.74 (0.29)	0.374

ABIC: Age-Bilirubin-INR-Creatinine Score; APRI: Aspartate Aminotransferase to Platelet Ratio Index; AST/ALT ratio: Aspartate Aminotransferase/Alanine Aminotransferase Ratio; BARD: Body mass index, AST/ALT ratio, Diabetes; BLR: Basophil-to-Lymphocyte Ratio; dNLR: Derived Neutrophil-to-Lymphocyte Ratio; ELR: Eosinophil-to-Lymphocyte Ratio; FIB-4: Fibrosis-4 Index; King score: King's College Hospital Score; LMR: Lymphocyte-to-Monocyte Ratio; Lok index: Lok Fibrosis Index; LogOddsLok: Log-Odds of Lok Index; MEF25%: Maximum Expiratory Flow at 25% of Forced Vital Capacity; NLR: Neutrophil-to-Lymphocyte Ratio; PLR: Platelet-to-Lymphocyte Ratio; PNR: Platelet-to-Neutrophil Ratio; RDW to PLT Ratio: Red Cell Distribution Width to Platelet Ratio; SII: Systemic Immune-Inflammation Index; TyG ratio: Triglyceride-Glucose Index.

Supplementary Table S4. Hemogram-derived ratios, hepatic steatosis, and liver fibrosis scores according to DLCO categories (Normal: >75% of predicted, up to 140%; Mild: 60-75%; Moderate: 40-60%; Severe: <40%)

**Variable**	**Normal (*n*=7)**	**Mild (*n*=17)**	**Moderate (*n*=20)**	**Severe (*n*=22)**	***p*-value**
NLR, median (IQR)	2.82 (1.51 - 4.79)	2.34 (1.82 - 4.08)	2.69 (2.3 - 4.12)	3.42 (2.96 - 4.52)	0.322
dNLR, median (IQR)	21.07 (3.61 - 24.73)	13.91 (7.82 - 18.48)	12.05 (9.06 - 27.51)	24.13 (16.42 - 33.37)	0.202
PLR, median (IQR)	125.9 (89.21 - 242.65)	156.2 (80.5 - 212.2)	118.78 (97.51 - 164.85)	138.57 (92.01 - 206.75)	0.965
LMR, median (IQR)	3.77 (2.01 - 4.6)	3 (2.15 - 5.04)	3.48 (2.61 - 5.19)	2.59 (1.93 - 3.71)	0.499
SII, median (IQR)	646.59 (301.14 - 1028.13)	723.46 (380.69 - 990.98)	577.29 (458.39 - 1087.35)	879.63 (605.52 - 1260.67)	0.459
PNR, median (IQR)	125.9 (89.21 - 242.65)	156.2 (80.5 - 212.2)	118.78 (97.51 - 164.85)	138.57 (92.01 - 206.75)	0.971
ELR, median (IQR)	0.1 (0.07 - 0.11)	0.09 (0.03 - 0.14)	0.11 (0.07 - 0.23)	0.16 (0.1 - 0.19)	0.221
BLR, median (IQR)	0.01 (0 - 0.02)	0.02 (0.01 - 0.03)	0.01 (0 - 0.02)	0.03 (0.01 - 0.03)	0.374
FIB-4, median (IQR)	1.46 (1.33 - 1.89)	1.49 (1.19 - 2.19)	1.6 (1.46 - 2.18)	1.47 (1.14 - 1.92)	0.769
APRI, median (IQR)	0.25 (0.18 - 0.33)	0.31 (0.19 - 0.37)	0.32 (0.2 - 0.44)	0.25 (0.19 - 0.42)	0.746
BARD, median (IQR)	3 (2 - 3)	3 (2 - 3)	3 (2 - 3)	3 (2 - 3)	0.929
AST/ALT ratio, median (IQR)	1.45 (1.11 - 1.54)	1.29 (1.08 - 1.57)	1.31 (0.88 - 1.9)	1.3 (1.12 - 1.58)	0.983
ABIC, median (IQR)	9.12 (8.75 - 9.2)	8.24 (7.53 - 8.43)	8.57 (7.85 - 8.72)	8.7 (7.81 - 8.88)	0.142
King score, median (IQR)	7.83 (6.06 - 9.21)	7.49 (4.65 - 10.57)	8.05 (5.83 - 11.78)	7.92 (6.54 - 11.42)	0.762
LogOddsLok, median (IQR)	-0.25 (-0.9 - 0.25)	-0.72 (-1.16 - 0.33)	-0.29 (-0.8 - 0.71)	0.41 (-0.31 - 1.5)	0.238
RDW TO PLT RATIO, median (IQR)	0.06 (0.05 - 0.07)	0.06 (0.04 - 0.07)	0.06 (0.04 - 0.07)	0.07 (0.05 - 0.08)	0.676
Lok index, mean (SD)	0.42 (0.16)	0.4 (0.22)	0.5 (0.25)	0.6 (0.3)	0.175
TyG ratio, mean (SD)	3.7 (0.2)	3.91 (0.22)	3.74 (0.26)	3.76 (0.2)	0.06721]

ABIC: Age-Bilirubin-INR-Creatinine Score; APRI: Aspartate Aminotransferase to Platelet Ratio Index; AST/ALT ratio: Aspartate Aminotransferase/Alanine Aminotransferase Ratio; BARD: Body mass index, AST/ALT ratio, Diabetes; BLR: Basophil-to-Lymphocyte Ratio; dNLR: Derived Neutrophil-to-Lymphocyte Ratio; DLCO: Diffusing Capacity for Carbon Monoxide; ELR: Eosinophil-to-Lymphocyte Ratio; FIB-4: Fibrosis-4 Index; King score: King's College Hospital Score; LMR: Lymphocyte-to-Monocyte Ratio; Lok index: Lok Fibrosis Index; LogOddsLok: Log-Odds of Lok Index; NLR: Neutrophil-to-Lymphocyte Ratio; PLR: Platelet-to-Lymphocyte Ratio; PNR: Platelet-to-Neutrophil Ratio; RDW to PLT Ratio: Red Cell Distribution Width to Platelet Ratio; SII: Systemic Immune-Inflammation Index; TyG ratio: Triglyceride-Glucose Index.

Supplementary Table S5. The association between pulmonary function tests and risk of advanced liver fibrosis assessed using FIB-4 score

**Variable**	**High (*n*=7)**	**Indeterminate (*n*=38)**	**Low (*n*=20)**	***p*-value**
FVC%, median (IQR)	70 (52 - 73.5)	75.5 (60.25 - 94.5)	69.5 (59 - 97.25)	0.49
FEV1%, median (IQR)	79 (61.5 - 83)	75 (58 - 100)	75 (60 - 98.5)	0.932
FEV1/FVC, median (IQR)	114 (111.5 - 114)	107 (99.25 - 110.75)	109.5 (101 - 114.25)	0.045
MEF75%, median (IQR)	65 (59 - 80)	84 (54.5 - 104.25)	81.5 (65 - 108.25)	0.694
MEF50%, median (IQR)	80 (67 - 90)	74.5 (51.75 - 94.25)	68 (58.5 - 92.5)	0.903
MEF25%, median (IQR)	63 (48 - 81)	54.5 (37.25 - 73.25)	55.5 (36.25 - 80.75)	0.668
DLCO, median (IQR)	44 (27.5 - 49.5)	51 (35.5 - 62.5)	47 (32.75 - 67.25)	0.365

DLCO: Diffusing Capacity for Carbon Monoxide; FEV1%: Forced Expiratory Volume in 1 Second Percent Predicted; FEV1/FVC: Forced Expiratory Volume in 1 Second Percent Predicted of Maximum Predicted/Forced Vital Capacity Percent Predicted; FIB-4: Fibrosis-4 Index; FVC%: Forced Vital Capacity Percent Predicted; MEF25%: Maximum Expiratory Flow at 25% of Forced Vital Capacity; MEF50%: Maximum Expiratory Flow at 50% of Forced Vital Capacity; MEF75%: Maximum Expiratory Flow at 75% of Forced Vital Capacity.

Supplementary Table S6. The association between pulmonary function tests and risk of advanced liver fibrosis assessed using APRI score

Variable	High (*n*=2)	Low (*n*=63)	Difference (95% CI)	*p*-value
FVC%, median (IQR)	79 (62 - 96)	70 (59.5 - 93)	9 (-57 - 61)	0.894
FEV1%, median (IQR)	76 (64 - 88)	75 (58 - 98.5)	1 (-51 - 44)	0.864
FEV1/FVC, median (IQR)	105.5 (101.25 - 109.75)	108 (101.5 - 113.5)	2.5 (-18 - 17)	0.909
MEF75%, median (IQR)	59 (54 - 64)	80 (59 - 106.5)	21 (-65 - 27)	0.279
MEF50%, median (IQR)	65 (62.5 - 67.5)	77 (57 - 93)	12 (-55 - 34)	0.662
MEF25%, median (IQR)	49 (47.5 - 50.5)	57 (39 - 80.5)	8 (-60 - 26)	0.648
DLCO, median (IQR)	38.5 (32.25 - 44.75)	48.5 (33.75 - 64.25)	10 (-46 - 21)	0.369

APRI: Aspartate Aminotransferase to Platelet Ratio Index; DLCO: Diffusing Capacity for Carbon Monoxide; FEV1%: Forced Expiratory Volume in 1 Second Percent Predicted; FEV1/FVC: Forced Expiratory Volume in 1 Second Percent Predicted of Maximum Predicted/Forced Vital Capacity Percent Predicted; FVC%: Forced Vital Capacity Percent Predicted; MEF25%: Maximum Expiratory Flow at 25% of Forced Vital Capacity; MEF50%: Maximum Expiratory Flow at 50% of Forced Vital Capacity; MEF75%: Maximum Expiratory Flow at 75% of Forced Vital Capacity.

Supplementary Table S7. The association between pulmonary function tests and risk of advanced liver fibrosis assessed using BARD score

Variable	High (*n*=60)	Low (*n*=5)	Difference (95% CI)	*p*-value
FVC%, median (IQR)	70 (59 - 93)	71 (61 - 113)	1 (-33 - 24)	0.631
FEV1%, median (IQR)	75 (57.75 - 97.75)	80 (66 - 100)	5 (-29 - 21)	0.758
FEV1/FVC, median (IQR)	108 (101.75 - 113.25)	112 (97 - 116)	4 (-11 - 11)	0.666
MEF75%, median (IQR)	79.5 (58 - 102.75)	75 (68 - 109)	4.5 (-40 - 30)	0.685
MEF50%, median (IQR)	75.5 (55.25 - 94.25)	61 (60 - 77)	14.5 (-20 - 32)	0.571
MEF25%, median (IQR)	55 (40 - 78.75)	74 (52 - 80)	19 (-32 - 20)	0.538
DLCO, median (IQR)	49 (32 - 64)	42 (40 - 51)	7 (-14 - 23)	0.799

BARD: Body mass index, AST/ALT ratio, Diabetes score; DLCO: Diffusing Capacity for Carbon Monoxide; FEV1%: Forced Expiratory Volume in 1 Second Percent Predicted; FEV1/FVC: Forced Expiratory Volume in 1 Second Percent Predicted of Maximum Predicted/Forced Vital Capacity Percent Predicted; FVC%: Forced Vital Capacity Percent Predicted; MEF25%: Maximum Expiratory Flow at 25% of Forced Vital Capacity; MEF50%: Maximum Expiratory Flow at 50% of Forced Vital Capacity; MEF75%: Maximum Expiratory Flow at 75% of Forced Vital Capacity.
